# Assessing coastal vulnerability at the village level using a robust framework, the example of Canacona in South Goa, India

**DOI:** 10.1016/j.isci.2024.109129

**Published:** 2024-02-13

**Authors:** Ritwik Nigam, Alvarinho J. Luis, Alexandre S. Gagnon, Eric Vaz, Bruno Damásio, Mahender Kotha

**Affiliations:** 1 School of Earth, Ocean and Atmospheric Sciences (SEAOS), Goa University, Taleigao Plateau, Goa 403206, India; 2 Division of Polar Remote Sensing, National Centre for Polar and Ocean Research, Ministry of Earth Sciences, Government of India, Headland Sada, Vasco-da-Gama, Goa 403804, India; 3 School of Biological and Environmental Sciences, Liverpool John Moores University, Liverpool L3 3AF, UK; 4 Department of Geography & Environmental Studies, Ryerson University, Toronto, ON M5B 2K3, Canada; 5 NOVA Information Management School (NOVA IMS), Universidade Nova de Lisboa, Campus de Campolide, Lisboa, Portugal

**Keywords:** Global change, Remote sensing, Hazard identification

## Abstract

Climate change poses a significant threat to coastal regions worldwide. This study presents and applies a modified Coastal Vulnerability Index (CVI) to assess coastal vulnerability at the village level, focusing on Canacona, a taluka in South Goa, India. It adapts the existing CVI methodology by incorporating additional variables to better represent the various dimensions of vulnerability, resulting in 21 variables split into a Physical Vulnerability Index (PVI) and a Social Vulnerability Index (SoVI). The results show spatial variability in coastal vulnerability across the studied villages, with Agonda and Nagercem-Chaudi found to be highly vulnerable and Loliem to be the least vulnerable. A hydrological modeling approach is also used to compare the CVI of every village with their susceptibility to inundation due to rising sea levels. The results demonstrate the influence of local factors on vulnerability, challenging previous taluka-level assessments given the scale upon which adaptation typically takes place.

## Highlights

• Focus on local coastal vulnerability, considering geological, physical, and social factors
• Study integrated 21 physical and socio-economic parameters
• World-renowned beaches face heightened vulnerability
• Social vulnerability parameters enhance the assessment of vulnerability

## Graphical abstract

**Figure:**
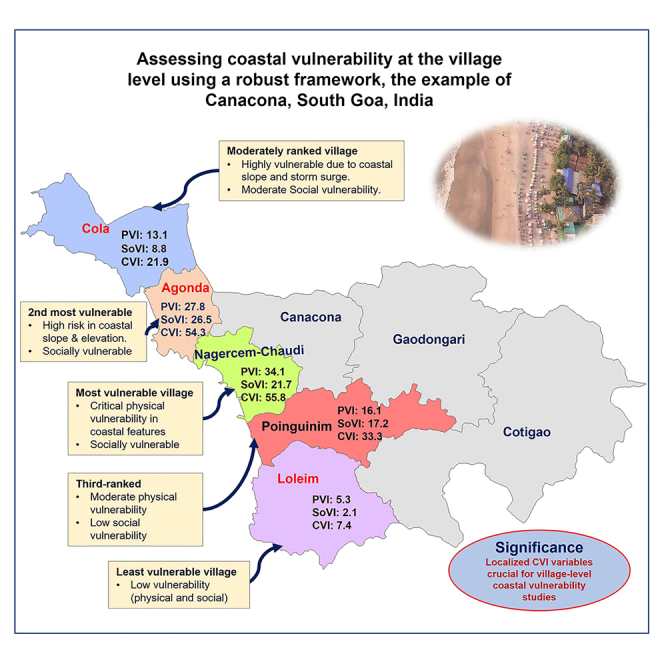

## Introduction

Climate change due to a continuous increase of greenhouse gases in the atmosphere is causing a rise in global temperature, sea surface temperature (SST), sea level rise (SLR), and changes in ocean currents, leading to gradual impacts on the coast.^1^ Moreover, many coastal areas are at risk of storm surges associated with tropical cyclones. This hazard could potentially increase, as warmer SSTs under climate change are likely to increase the frequency and/or intensity of tropical cyclones.^2^ When compounded with a higher sea level due to the thermal expansion of the oceans and melting of the cryosphere, this would cause abrupt impacts on the coast, further increasing the risk to low-lying coastal areas. In September 2019, the Intergovernmental Panel on Climate Change (IPCC) estimated that global sea levels would increase by between 61 cm and 1.1 m by the end of the century,^1^ a projection that is 10 cm higher than the figure reported in the latest IPCC report published in 2013, but which remains conservative considering the SLR of 1.49 m by 2100 previously reported by Rahmstorf.^3^ Furthermore, several developing countries, including India, are experiencing rapid urbanization, causing land use and land cover changes, particularly in coastal regions. It is estimated that around 40% of the world’s population now lives in coastal areas (United Nations, 2017), with projections indicating that these areas will continue to experience greater population growth than inland locations.^4^

Goa, the smallest state in India, is experiencing rapid urbanization, which increased from approximately 49% to 62% during the ten years preceding the 2011 census.^5^ One reason for this drastic increase in the urban population is that the state is a popular destination for national and international tourists (Govt. of Goa, 2016) given its abundance of beaches, national and international tourists (Govt. of Goa, 2016) given its abundance of beaches, which adds pressure for development and recreational activities along its 105 km long coastline. In addition, SLR, coupled with the effects of storms and their associated surges, is increasing coastal vulnerability, causing flooding and inundation of low-lying areas. This is particularly of concern to coastal areas such as sandy beaches, estuaries, and floodplains, which are of significant recreational and/or ecological value.^6^ Assessing the risk of these environments to coastal hazards and SLR is required to inform local adaptation. However, current assessments of vulnerability are not yet available at the spatial resolution necessary for adaptation, as they are mainly limited to ranking the administrative units of a state, known as talukas, in order of their risk to SLR, and this, mainly based on physical and geological parameters.^7^ Assessments of vulnerability at a higher spatial resolution are important for coastlines composed of highly varied landforms such as the state of Goa.

Most coastal vulnerability assessments have to date been limited to physical and geological parameters, plus vegetation Pantusa et al.^8^ The vulnerability of the coast to SLR or any other coastal hazard is not only a function of its susceptibility to geological parameters (i.e., rate of shoreline change, coastal regional elevation, coastal slope, geomorphology, and beach width), physical processes (i.e., rate of sea-level change, tidal range, and significant wave height) and its exposure to short-term hazards such as storm surges and tsunamis, but also contextual or social vulnerability. Social vulnerability refers to the socio-economic and demographic factors affecting the resilience of communities,^9^ as this influences the capacity for adaptation. According to Boruff et al.,^10^ these factors and the coast's physical and geological characteristics must be included for a thorough vulnerability assessment.

Assessing vulnerability at the village scale by integrating physical and social variables would provide baseline data to policy-makers to inform development plans at state level and for coastal adaptation at the community level. Accordingly, this paper presents an index-based methodology consisting of 21 variables for assessing coastal vulnerability at the local level, and applies it to the taluka (sub-district headquarters) of Canacona in South Goa district, India. Canacona was selected as the study location because its highly varied coastal topography provides an excellent example of spatial variation in vulnerability and because increasing tourism activities often lead to hazardous development in the low-lying villages in proximity to sandy beaches.

## Study area

**Figure 1:**
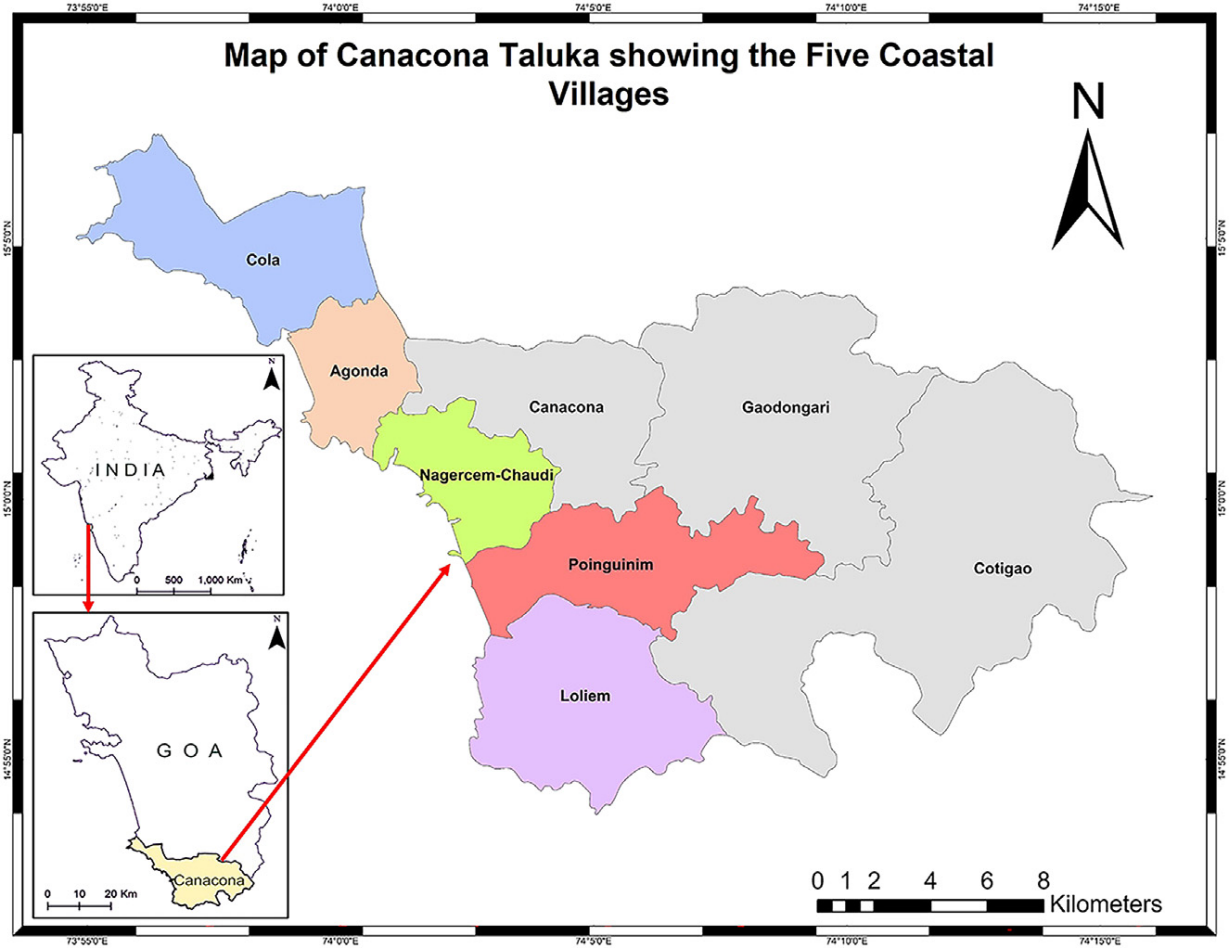
Map of the study area showing the location of Canacona taluka in the South Goa, India

Canacona is bordered to the north by Quepem taluka and to the northeast by the taluka of Sanguem, both of which are within the state of Goa (Figure 1). The state of Karnataka borders the southern part of Canacona, and the Arabian Sea is found to the West, and the Arabian Sea is found to, and the Arabian Sea is found in the west. Canacona taluka consists of eight villages, with five of them situated on the coast, i.e., Cola, Agonda, Nagercem-Chaudi, Poinguinim, and Loliem (Figure 1), which form the focus of this study. The coastline of Canacona consists of varied topographic forms, including sandy beaches, estuaries, headlands, and islands. The famous sandy beaches of Agonda, Palolem, and Patnem are in the study area.

## Methodology

**Figure 2:**
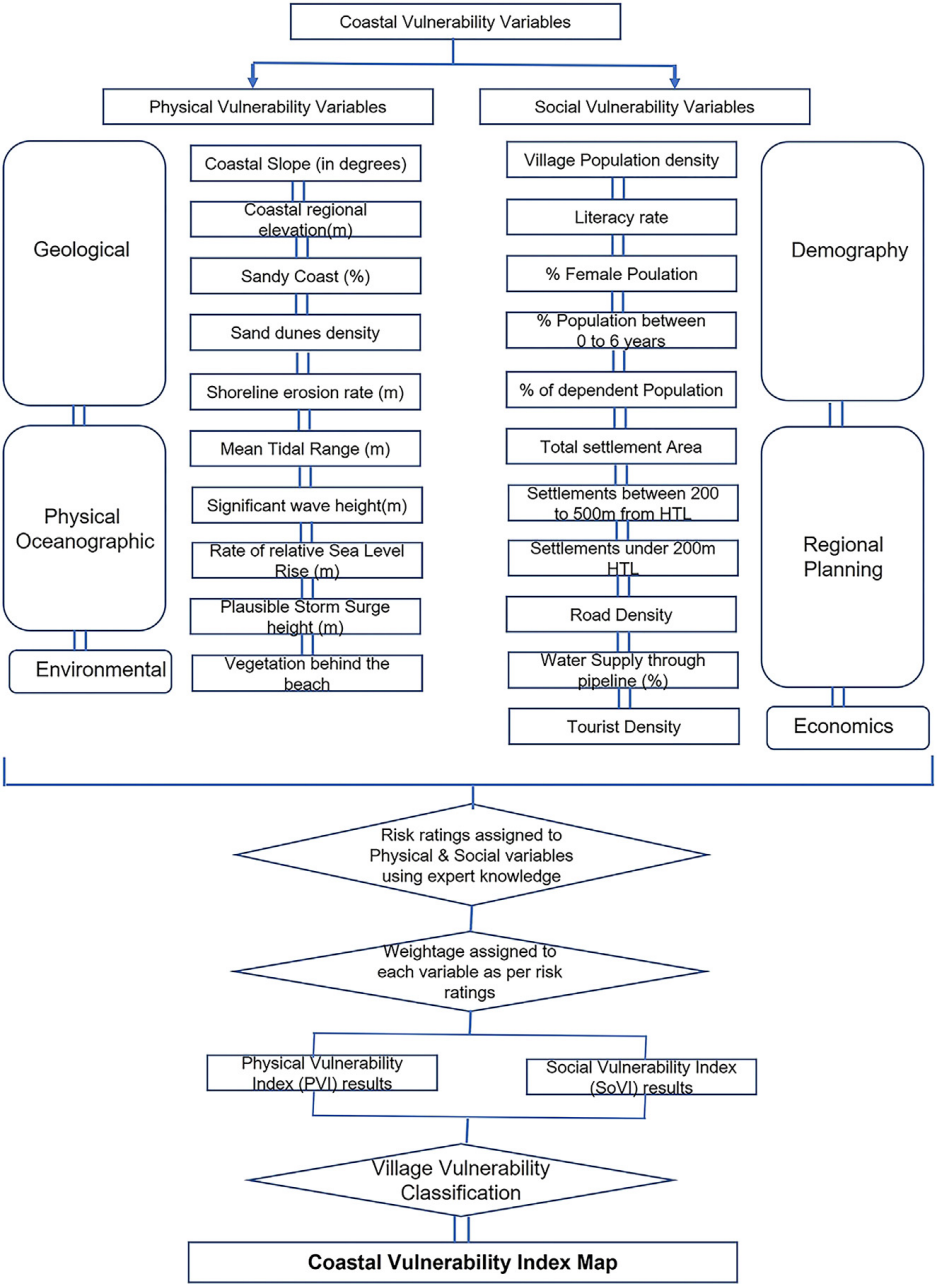
Flowchart showing robust framework designed to compute CVI

This study uses a modified version of the CVI methodology (Figure 2) first published by the United States Geological Survey (USGS),^11^^,^^12^^,^^13^ which assesses coastal vulnerability based on six physical and geological variables. In the current study, a Physical Vulnerability Index (PVI) is computed using ten variables (Table 1), including the six variables from the original USGS index, i.e.: rate of relative sea-level change, rate of shoreline change (erosion or accretion), mean tidal range, significant wave height, coastal slope, and geomorphology, plus coastal regional elevation and other variables that have not yet been incorporated in CVI along the coast of India, that is the percentage of sandy coast, dune density and vegetation behind the beach, or their use has yet been limited: plausible storm surge height. These latter three variables were added because local morphological factors and vegetation can influence the degree of vulnerability. The Social Vulnerability Index (SoVI), for its part, comprises 11 social variables (Table 2); Village population density, road density, total settlement area under village (%), water supply through pipeline (%), literacy rate (%), dependent population (%), female population (%), population between 0–6 years (%), tourist density, settlement area under 500m HTL, and settlement area under 200m HTL.

**Table 1:** Physical variables significance and source with respect to coastal vulnerability

S No.	Variable	Derived by	Significance	Source	Year	Ground Truth
1	Coastal slope (degree) (Figure 3)	Referred to CVI of Goa coast by Kunte et al.	Locations with gentle slope are more prone to flooding as gentle slopes retreat faster than steeper slope and wave energy can penetrate far inland due to less frictional force restricting it.	ETOPO2 bathymetric dataset from the NIO, Goa	1971–1984	The devastating Indian Ocean tsunami in 2004 profoundly impacted the coastal city of Banda Aceh, Indonesia. The coastal areas with gentle slopes were more vulnerable to the powerful tsunami waves that penetrated far inland, causing widespread destruction. The lack of natural barriers and the flat terrain allowed the waves to travel farther inland, causing more extensive flooding.
2	Coastal regional elevation (m)	SRTM 30m DEM, using QGIS	Coastal regional elevation is an important parameter as it provides an estimate of the extent of land threatened by projected SLR (Kumar and Kunte, 2012; Kumar et al., 2010^14^), and the coast's sensitivity to flooding during a storm surge^15^ or tsunami. Hence, areas with high coastal elevation will be considered less vulnerable and vice-versa.^16^	SRTM 30 m DEM	2019	The 2011 Tōhoku earthquake and subsequent tsunami in Japan highlighted the importance of coastal elevation in tsunami vulnerability. For example, the city of Aonae in the Miyagi Prefecture suffered less severe impacts due to its elevated location on a hillside.
3	Sandy coast (%)	Total sandy coast length in each village/Total coastline length of each village x 100.	Sandy coasts have gentle slope toward inland which makes their inland areas more vulnerable to coastal flooding. Higher the amount of sandy coast in a given transect higher will be the vulnerability.	Google Earth and Digimizer	2019	Hurricane Sandy, had a profound impact on the northeastern United States. New Jersey was severely affected. It has a significant stretch of sandy coastline with relatively gentle slopes. During Hurricane Sandy, the storm surge inundated large portions of the coast, flooding coastal communities and causing extensive damage. The combination of the storm surge, high tide, and the gentle slope of the sandy coast contributed to the widespread flooding.
4	Dune density	Dune areas from the NCSCM study were digitized and measured using Digimizer software	Sand dunes protect the inland area from seawater intrusion during high tides and act as a reservoir for future supplies to maintain the beach. Sparse sand dune areas are more vulnerable as there is no restriction between the sandy beach and inland areas.	NCSCM 2017, Field Study	Oct, 2019	The Dutch coast along the North Sea features an extensive sand dunes system critical in protecting the low-lying coastal areas from flooding and erosion.
5	Vegetation behind the beach	Vegetation areas in the imageries were measured using Digimizer software.	Casaurina and coconut plantation help decrease the energy from cyclonic winds and storm waves, reducing the risk of inland damage. Dense vegetation could lessen the vulnerability	Field survey, Google Earth Imageries and Digimizer	Sept 2019	Chennai, India, has been vulnerable to the impacts of cyclones and storm surges. In response, the Tamil Nadu Forest Department established casuarina plantations along vulnerable stretches of the coastline. These casuarina plantations act as windbreaks and help protect coastal communities from the destructive forces of cyclonic winds and storm waves.
6	Rate of relative SLR (m)	Adopted from the CVI of Goa coast by Kunte et al.	A higher SLR increment rate will increase the vulnerability as it denotes increasing the risk of inundation in low-lying coastal areas.	Annual mean relative sea level data from Indian Ocean tide gauge stations 1969–2017.	1969–2017	Bangladesh is a densely populated country with a significant portion of its population residing in low-lying coastal areas. The combination of higher sea-level rise rates and flat topography makes these areas particularly vulnerable to coastal inundation, which can lead to community displacement and loss of agricultural land.
7	Shoreline erosion rate (m)	Adopted from Indian Shoreline Atlas, 2018	Shoreline erosion rate over some time indicates the sensitivity of the coast, thus increased vulnerability.	NCCR, MoES 2018	2018	The Chesapeake Bay region has experienced significant shoreline erosion due to various factors, including sea-level rise, wave action, and human activities. Erosion along the bay’s coastlines has led to the loss of valuable land, including residential properties and infrastructure.
8	Mean tidal range (m)	Data from the National Institute of Oceanography, Goa	Coastal areas with high tidal range and low coastal regional elevation are highly vulnerable.	NIO, Goa India, 2011	2011	Coastal regions along the Bay of Bengal in India, such as parts of West Bengal and Odisha, experience high tidal ranges due to the bay’s unique geography. Low-lying coastal areas in these regions are particularly susceptible to cyclones and storm surges, leading to flooding, erosion, and damage to homes and infrastructure.
9	Significant wave height (m)	Data from the National Institute of Oceanography, Goa	The average height (trough to crest) of one-third of the waves in a wave spectrum for a given period.^14^ Dwarakish et al. (2009) considered significant wave heights range from 1.6 to 2.8 m as moderately vulnerable.	NIO, Goa, India using studies on directional waves off Mormugao Port, 2009	2009	Coastal regions along the Bay of Bengal, such as parts of Bangladesh, India, and Myanmar, are exposed to high significant wave heights and storm surges during cyclones. These factors leads to extensive coastal flooding, erosion, and damage to homes and infrastructure.
10	Plausible storm surge height (m)	Derived from Vulnerability Atlas of India, 2019	Due to its high-intensity waves, storm surges can destroy low- lying coastal areas. Thus, surge height in any region is an important criterion.	BMTPC, Ministry of Home and Urban Affairs	2019	The surge height associated with Hurricane Katrina reached approximately 28 feet (8.5 m) in some areas along the Gulf Coast. The storm surge inundated large portions of low-lying coastal regions in Louisiana, Mississippi, and Alabama, causing catastrophic flooding, destruction of infrastructure, and loss of life. The surge height played a central role in the unprecedented devastation experienced by the affected communities.

**Figure 3:**
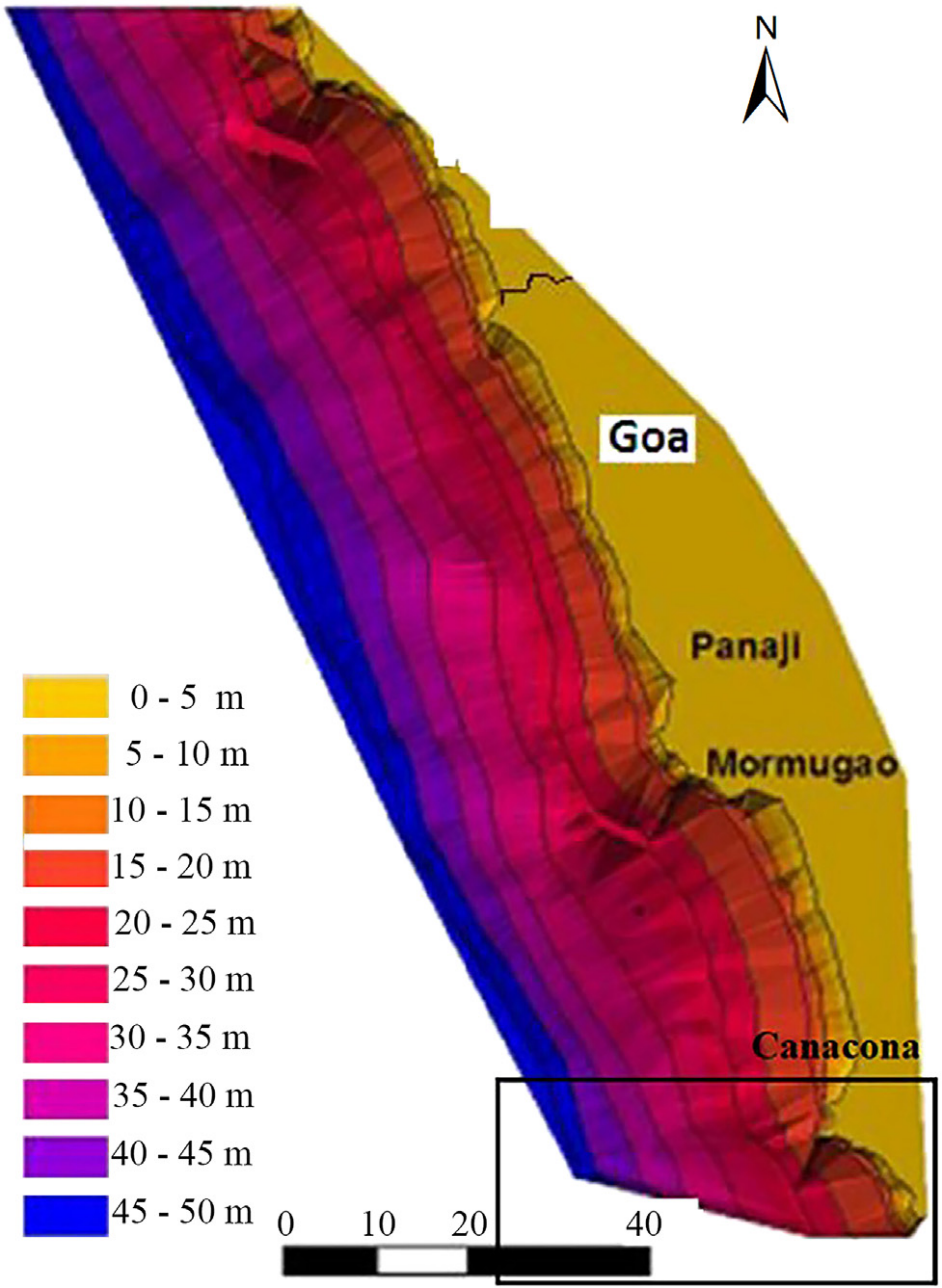
Nearshore bathymetry of the Goa coast, Canacona bathymetry shown in the box (down right)

**Table 2:** Showing social variables significance and source with respect to coastal vulnerability

S No.	Variable	Derived using	Significance	**Source**	Year	Ground Truth
1	Village population density	Total population/Total village area (Sq. Km) x 100	High population density in a region will lead to higher vulnerability due to high pressure on available resources	Census of India	2011	Tokyo Bay is home to Tokyo, one of the most populated cities in the world. The city high population density and coastal location makes it vulnerable to both storm surges and tsunamis. The 2011 Tōhoku earthquake and tsunami, which led to the Fukushima nuclear disaster, highlighted the risks posed by tsunamis to densely populated coastal areas.
2	Road density	Measured road length from regional planning map using Digimizer and calculated Total road length/100 Sq. km for each village	Low road density will lead to high vulnerability as evacuation and assistance will be more difficult during an emergency.	Regional planning map of Canacona, 2021 and Digimizer	2019	The Andaman and Nicobar Islands, located in the Bay of Bengal, were severely impacted by the 2004 Indian Ocean tsunami. The low road density on some of these islands hampered the immediate response and aid distribution efforts. Evacuation routes and access to affected areas were limited, delaying rescue operations and hindering the delivery of essential supplies.
3	Total settlement area under village (%)	Digitized and measured regional planning map of Canacona taluka using Digimizer software.	The higher the settlement area under a coastal village, the higher the exposure to coastal hazards, thus, the higher the vulnerability.	Regional planning map of Canacona, 2021 and Digimizer	2019	Palu, Indonesia, experienced a devastating tsunami in 2018. The extensive settlement area along the coast increased exposure to the tsunami hazard. The high population density and significant infrastructure within the settlement area exacerbated the impacts, leading to widespread destruction and loss of life.
4	Water supply through pipeline (%)	No. of metered connection/Total village population x 100	Households without metered water connection extract groundwater, could become vulnerable during salt water intrusion due to a coastal hazard	Statistical Handbook of Goa	2016–17	Cyclone Sidr in 2007, in Bangladesh had caused widespread devastation. Many households relied on shallow tube wells for freshwater, lacking metered water connections. The cyclone’s storm surge led to saltwater intrusion into groundwater sources, rendering the well water undrinkable. This left households without access to safe drinking water, exacerbating the dire situation after the disaster.
5	Literacy rate	Total literate population/Total population x 100	A higher % of literacy suggests better decision making capacity during a hazard, hence low vulnerability	Census of India	2011	Cyclone Phailin struck Odisha, India, in 2013. The Indian government’s efforts to promote literacy and disaster preparedness played a crucial role in reducing vulnerability. As a result, despite the cyclone’s intensity, many people were successfully evacuated, significantly reducing casualties and mitigateing the impact on coastal communities.
6	Dependent population (%)	Total village population - Total working population (14–45 years) = non- working population Then, Total non-working population/Total village population x 100 = Dependent population in %	The dependent population is always at the highest risk, which can decrease a non-dependent person efficiency during an emergency	Census of India	2011	The aftermath of Hurricane Katrina highlighted the vulnerabilities of dependent populations. Many elderly residents, individuals with disabilities, and those with limited mobility were unable to evacuate before the storm hit. The lack of accessible transportation, shelters, and proper support networks led to tragic outcomes for these individuals.
7	Female population (%)	Total female population/Total village population x 100	Generally, a low literacy rate and a high malnutrition rate make them dependent population during the time of an emergency.	Census of India	2011	The Indian Ocean tsunami in 2004 disproportionately affected women in vulnerable coastal communities. Low literacy rates and cultural norms restricted their ability to seek safety and access information. Women, especially those who were pregnant or caring for young children, faced challenges in evacuating and accessing resources.
8	Population between 0–6 years (%)	Under six years population/Total village population x 100	Illiteracy and lack of physical efficiency make the population under six years dependent and highly vulnerable.	Census of India	2011	Hurricane Katrina, 2005 highlighted the vulnerabilities of a population with a significant number of children. The evacuation process was complicated by ensuring the care and safety of children, and overcrowded shelters further increased the difficulties faced by families
9	Tourist Density (Person/Sq. km)	Total tourists visited/Total village population x 100	A high tourist population in a coastal village will increase their exposure and add pressure on available resources during a coastal hazard.	Field observation and Directorate of Tourism, Goa data	2016–17	The presence of tourists on the island added complexity to emergency response and recovery efforts when Hurricane Maria struck Puerto Rico in 2017. Tourists faced challenges in accessing timely information and evacuating. Also, the strain on resources and infrastructure impacted the overall ability to respond effectively to the disaster.
10	Settlements between 200m and 500m from HTL	Settlement areas under 500m HTL shown in the regional planning map of Canacona were digitized and measured using Digimizer.	Due to high exposure to coastal hazards, this zone cannot have high concentration of settlement areas.	Regional plan of Canacona, Goa, T And CP, Goa		India’s Coastal Regulation Zone (CRZ) is a comprehensive set of regulations to protect the coastal environment and reduce vulnerability to hazards. The CRZ framework designates specific zones along the coast with varying degrees of restrictions on development activities, including settlements, within certain distances from the high tide line.
11	Settlements under 200m HTL	Settlement areas under 200m HTL shown in regional planning map of Canacona were digitized and measured using Digimizer.	According to CZMP 2019, the CRZ-3B zone cannot have any permanent settlement development under 200m from High tide line. They are most exposed and vulnerable.	Regional plan of Canacona, Goa, Town and country planning, Panaji, Goa		India’s Coastal Regulation Zone (CRZ) is a comprehensive set of regulations to protect the coastal environment and reduce vulnerability to hazards. The CRZ framework designates specific zones along the coast with varying degrees of restrictions on development activities, including settlements, within certain distances from the high tide line.

Different methods are used to determine the values of the variables comprising an index. The USGS methodology^11^^,^^12^^,^^13^ ranks each variable on an ordinal scale from one to five. Most studies, worldwide, have followed this ranking procedure, but without necessarily using the same range for the ranking of the risk variables as no standards exist to determine what values should be considered as low, medium, or high when a three-category scale is used, for instance, nor is it the case when five ranking categories are used. Boruff et al.,^10^ following the USGS methodology, ranked the variables as low, moderate, and high, and, in the same way, Kumar and Kunte (2012) classified the coastline of Chennai into three vulnerability categories. Likewise, this study follows this three vulmerability category (Tables 3 and 4). All 21 variables were ranked with the help of expert knowledge. The ranking score of three physical variables (rate of relative SLR, mean tidal range, and significant wave) is taken from Kunte et al.,^7^ as changes in those variables along the 42-km study area of Goa coastline are marginal. Sand dune density and vegetation behind the beach along the Canacona coastline were ranked qualitatively based on the field observations taken by experts. The ranking of each of the 21 variables (Tables 5 and 6) was used to calculate the indices as per the procedure described below:

**Table 3:** Risk ratings assigned to 10 physical variables (using available literature and expert knowledge)

S. No.	Physical Variables	Low (1)	Medium (2)	High (3)
1	Coastal slope (Degree)	>0.3	0.1–0.3	<0.1
2	Coastal regional elevation (m)	>50	20–50	<20
3	Sandy coast (%)	<10	10–20	>20
4	Dune density	Rocky coast	Moderate	Sparse
5	Vegetation behind the beach	Dense	Moderate	Sparse
6	Rate of relative SLR (m)	–	1.29	–
7	Shoreline erosion rate (m)	<0.3	0.3–0.6	>0.6
8	Mean tidal range (m)	–	0.2–2.4	–
9	Significant wave height (m)	–	0.6–2.0	–
10	Plausible storm surge height (m)	–	–	4.5

**Table 4:** Risk ratings assigned to the 11 social variables (using available literature and expert knowledge)

S. No.	Social Variables	Low (1)	Medium (2)	High (3)
1	Village population density	<100	100–200	>200
2	Road density	>100	50–100	<50
3	Total settlement area under village (%)	<15	15–30	>30
4	Water supply through pipeline (%)	>80	40–80	<40
5	Literacy rate (%)	>80	75–80	<75
6	Dependent population (%)	<30	30–60	>60
7	Female population (%)	<30	30–60	>60
8	Population between 0–6 years	<5	5–10	>10
9	Tourist density (Person/Sq. km)	<100	100–200	>200
10	Settlements under 500m HTL (%)	<10	10–20	>20
11	Settlements under 200m HTL (%)	–	–	>5

**Table 5:** Risk ranking assigned at the village level for every variable comprising the PVI

VILLAGE →VARIABLES	Cola	Agonda	Nagercem-Chaudi	PoinguInim	Loliem
Coastal slope (Degree)	3	3	3	3	3
Coastal regional elevation (m)	2	3	3	3	1
Sandy coast (%)	2	2	3	3	1
Sand dune density	1	3	3	2	1
Vegetation behind the beach	1	3	3	3	1
Rate of relative SLR (m)	2	2	2	2	2
Shoreline erosion rate (m)	1	2	2	1	1
Mean tidal range (m)	2	2	2	2	2
Significant wave height (m)	2	2	2	2	2
Plausible storm surge height (m)	3	3	3	3	3

**Table 6:** Risk ranking assigned at the village level for every variable comprising the SoVI

VILLAGE → VARIABLES	Cola	Agonda	Nagercem-Chaudi	Poinguinim	Loliem
Village population density	2	3	2	2	2
Road density	3	2	2	2	3
Total settlement area under village (%)	1	3	2	1	1
Water supply through pipeline (%)	1	1	1	1	1
Literacy rate (%)	2	2	2	2	1
Dependent population (%)	2	3	2	3	2
Female population (%)	2	2	2	2	2
Population between 0–6 years	3	3	3	2	2
Tourist density (Person/Sq. Km)	2	3	3	1	1
Settlements under 500m HTL (%)	3	2	3	3	1
Settlements under 200m HTL (%)	1	2	3	2	1

The PVI and SoVi were calculated as the squared root of the product of the ranked variables divided by the number of variables as described in USGS (1999):

(Equation 1) PVI=a1∗b1∗c1∗d1∗e1∗f1∗g1∗h1∗i1∗j110

where a_1_ = coastal slope, b_1_ = coastal regional elevation, c1 = percentage of sandy coast, d1 = sand dunes density, e1 = vegetation density behind the beach, f1 = rate of relative SLR, g1 = shoreline erosion rate, h1 = mean tidal range, i1 = significant wave height, j1 = plausible storm surge height.

In their study of part of Maharashtra coast to assess coastal vulnerability by using CVI methodology Sharma and others pointed out that in the previous studies, the coastal slope is used as one of the parameters for calculating the CVI, with low coastal slope representing high risk and vice versa. Such an assumption is not always correct. For example, areas with low coastal slopes falling in areas of high coastal regional elevation are not as vulnerable as similar areas falling in low coastal regional elevations. The coastal regional elevation could also be considered an additional parameter representing the vertical level of the terrain. An extensive account of the various studies on the coastal vulnerability assessment of other areas is included in Kantamaneni et al. (2019).^17^The SoVi was calculated as follows:

(Equation 2) $$\text{SoVI}=\sqrt{a2*b2*c2*d2*e2*f2*g2*h2*i2*k2/11}$$

where a2 = village population density, b2 = road density, c2 = total settlement area under village (%), d2 = water supply through pipeline (%), e2 = literacy rate, f2 = dependent population, g2 = female population (%), h2 = population between 0–6 years, i2 = tourist density, j2 = settlements under 500m High Tide Line (HTL), and k2 = settlements under 200m HTL.

Then, the sum of the PVI and SoVi is calculated to determine the CVI, i.e.,

(Equation 3) CVI=PVI+SoVI

The present study has adopted a similar scale implemented by Kunte et al.^7^ who also assessed similar coastal stretches but at a much coarser spatial scale (at the taluka level). The objective behind using a similar scale for CVI values is to compare the results of the current study (at the village level) with Kunte et al.^7^ to highlight the importance of a high spatial resolution CVI compared to a low spatial resolution CVI.

## Results

**Figure 4:**
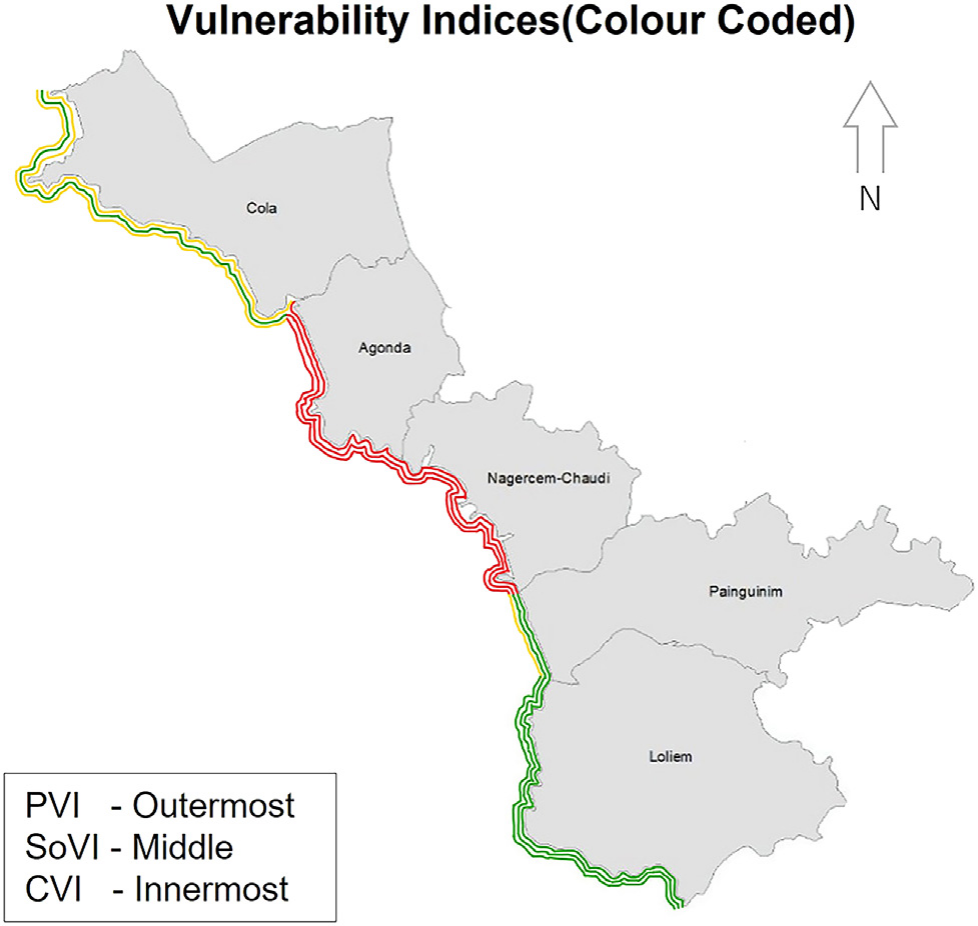
Village map showing computed results of PVI, SoVI, and CVI

**Figure 5:**
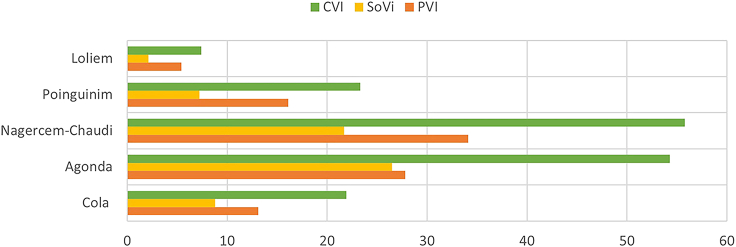
Results of the three indices for each village

**Figure 6:**
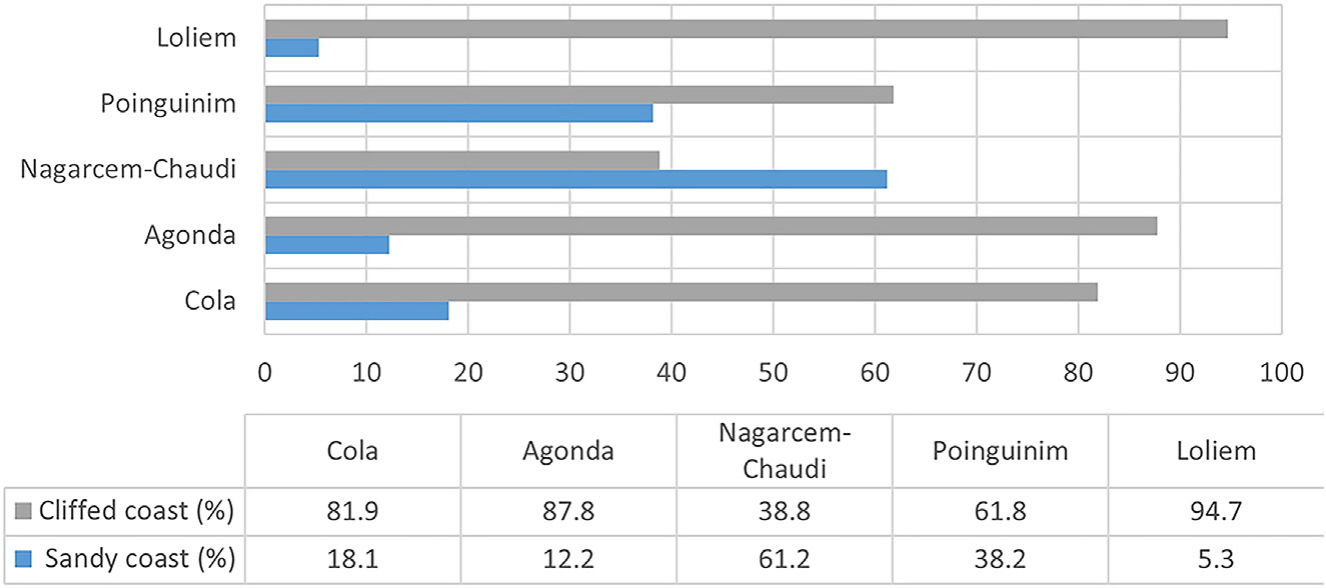
Ratio of sandy coast with gentle inland coast and rocky cliff in each village

The vulnerability index scores and graphical illustration of the results are presented in (Figures 4, 5, and 6) and (Table 7). The categorization of the CVI values in Table 7 was adopted from.^7^ The following paragraphs detail the variation in the indices and the reasons explaining the corresponding index values.

**Table 7:** PVI, SoVI, and CVI results

Coastal vulnerability index			
Village	PVI	SoVI	CVI
Cola	13.1	8.8	21.9
Agonda	27.8	26.5	54.3
Nagercem-Chaudi	34.1	21.7	55.8
Poinguinim	16.1	7.2	23.3
Loliem	5.4	2.1	7.5
**CVI Values**	**<10 (Low)**	**10-20 (Moderate)**	**>20 (High)**

### Cola:

With a PVI of 13.1, Cola is ranked as moderately vulnerable. The high-risk physical variables were found to be coastal slope (<0.1°) and plausible storm surge height (4.5 m). These two variables are uniform in the five villages of the study area. The proportion of sandy coast, the rate of relative SLR, mean tidal range, and significant wave height fall in the moderate risk category. Sand-dune density and vegetation behind the sandy beach resulted in this village being assigned in the low-risk category for those two variables. Moreover, a significant portion of the coastline is cliffed (81.9%, from Cabo de Rama Fort to Cola-Agonda village border), which makes it the second least physically vulnerable village in the study region. For its part, social vulnerability lies in a low to moderate range (SoVI = 8.8). Except for low road density, a high proportion of the population under six years of age, and settlement under 500m of HTL, all other variables have low or moderate risk values. This village has recently had new seaside holiday resorts with limited road connectivity. When put together, the PVI and SoVI values give a CVI value of 21.9 for this villlage, the second lowest in the study region.

### Agonda

Agonda was found to be the second most vulnerable village with the second-highest PVI (27.8), the highest SoVI (26.5), and second-highest CVI (54.3). The physical variables with the highest risk ratings were low coastal slope (<0.1°) and coastal regional elevation, as the latter is less than 50m, on average, with many parts of this village extensively low-lying. Moreover, the sand dune density and the vegetation behind the beach are in the hig risk category. The pressure of tourist activities and haphazard development on a long stretch of Agonda beach has destroyed dunes and vegetation behind the dune. Furthrmore, the chances of a storm surge that is 4.5m high add to the vulnerability. Despite a good road network (100), high literacy rate (>80%), water supply through pipelines (>80%), and a small number of settlements under 200m and 500m from HTL (<10%), Agonda has the highest social vulnerability. The reason behind this is very high population density (>200), high tourist density (>200), and a high percentage of dependent population (>60%) along the low-lying estuarine and coastal belt. These socio-economic variables, in addition to physical variables, make Agonda a highly vulnerable coastal village.

### Nagercem-Chaudi

The study found Nagercem-Chaudi, the most vulnerable village, with very high PVI (34.1), SoVI (21.7), and CVI (55.8) values. An extensive long sandy coastline (61.2%) with encroached and degraded cliffs and world-renowned beaches (i.e., Palolem and Patnem) make Nagercem-Chaudi village the hub of tourist activity in South Goa. It is evident from the values of coastal slope (<0.1°), coastal regional elevation (<20m), sandy coast (61.2%), sand-dune density (least), and vegetation behind the beach (least) that this village is highly vulnerable. Nagercem-Chaudi is also the administrative headquarters of Canacona taluka, making it socially vulnerable. Demographic variables such as high population density (>200), high settlement concentration under 200m and 500m above HTL (>20%), and high tourist density (>200) give this village the highest vulnerability ranking of the fice study villages. Nagercem-Chaudi has seen haphazard development due to the high concentration of tourist activities without broad roads, mainly near the coastal belt. It is extensively located between low-lying agricultural fields, leading to a high degree of vulnerability far inland.

### Poinguinim

Poinguinim was ranked third among the five villages with PVI (16.1), SoVI (17.2), and CVI (33.3), respectively. The topography of the village is similar to that of Nagercem-Chaudi. The beaches of Rajbagh and Galgibaga are located in the village. Physical variables such as coastal slope (<0.1°), coastal regional elevation (<20 m), and sand-dune density (least) were found to be highly vulnerable; all other variables were ranked moderate and low. On the other hand, Poinguinim ranked low in social vulnerability; the exceptions to this were a highly dependent population (>60%) and a high number of settlements under 500m from HTL (>20%), one of the highest among all villages. The village has not developed tourism compared to its adjacent Agonda and Nagercem-Chaudi. Due to te golf course, beach morphological modification has occurred recently, mainly at Rajbagh beach.

### Loliem

Loliem ranked as the least vulnerable village in Canacona with PVI (5.3), SoVI (2.1), and CVI (7.4). It was ranked moderate to low for almost all the variables, except those that were considered uniform for the whole study area. The primary factor behind its low vulnerability is the predominantly cliffed coast (94.7%) and high regional elevation (>50m). These two have extensively influenced the low degree of vulnerability that could have been maximized due to other physical and social variables. The only notable exception is low road density (100), which could add to the vulnerability of adjacent villages.

## Validation

To validate the CVI results, a hydrological modeling approach was implemented to assess the coastal vulnerability associated with potential coastal hazards.

The study utilized a modified “bathtub” model in conjunction with local digital elevation models (DEMs) to outline the areas susceptible to future SLR inundation. In contrast to the conventional “bathtub” model, which assumes that land elevation below projected sea levels will be submerged, the modified model incorporates hydrological connectivity (HC). This feature enables the model to exclude areas disconnected from open water from inundation.

As depicted in the figure below, the study considered two types of HC: the “four-sided” and “eight-sided” rules. The “four-sided” rule considers a grid cell in the DEM hydrologically connected if its cardinal directions are linked to a flooded cell. On the other hand, the “eight-sided” rule assumes connectivity in both cardinal and diagonal directions. The study opted for the eight-sided rule to represent real-world conditions more accurately.

Three plausible compound coastal flooding inundation scenarios, consisting of SLR, Higher High Water at Spring (HHWS), and Storm Surge (SS) were used to determine the extent of inundation along the coast, which is directly proportional to the coastal vulnerability in the respective area. The 30-m freely available SRTM DEM was retrieved from the NASA Earth Explorer for the study area (see Tables 8 and 9).

**Table 8:** Three plausible compound coastal inundation scenarios and their inundation depths

	Scenario elements	Source	Inundation depth (m)	Total inundation depth
1	SLR-1 + HHWS + SS	IPCC AR-6, MPT & BMTPC, 2019	0.55 + 1 + 4.5	6.05
2	SLR-2 + HHWS + SS	IPCC AR-6, MPT & BMTPC, 2019	1.02 + 1 + 4.5	6.52
3	SLR-3 + HHWS + SS	IPCC AR-6, MPT & BMTPC, 2019	1.61 + 1 + 4.5	7.11

**Table 9:** Land use land cover changes in the study area between 2000 and 2020

Study area	Built-Up	Agriculture
Coastal Canacona (2000)	0.598	1.832
Coastal Canacona (2020)	1.098	2.005
Increment (%)	83.61	9.4

**Figure 7:**
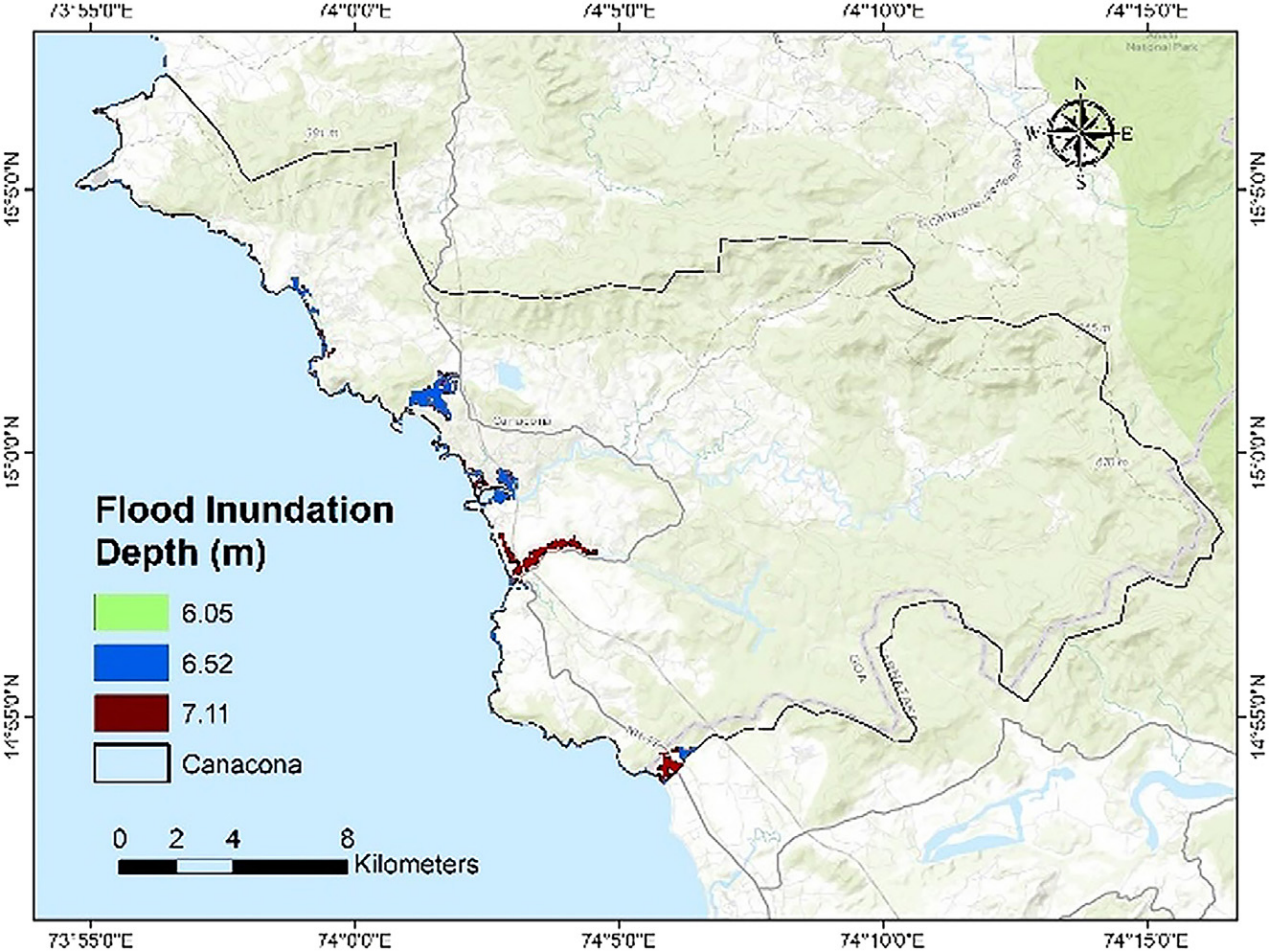
Flood inundation depth map

The evaluation of SLR-engendered inundation in the study area highlights that villages with higher Potential Vulnerability Index (PVI) scores, namely Agonda, Palolem, and Poinguinim, are particularly susceptible to coastal flooding (as illustrated in Figure 7; Table 8), mainly due to the presence of estuaries within these villages. Moreover, the noticeable increase in built-up and agricultural areas strongly indicates a corresponding rise in developmental activities along the coastal belt, aligning with the study area's SoVI values. Therefore, the congruent findings from the CVI and SLR assessments suggest that villages with higher CVI values face a greater risk of coastal hazards than those with lower CVI values.

## Discussion

The present study has introduced a robust CVI framework that is practical at the grassroots level by incorporating 21 physical, geological, and socio-economic variables, which have never been adopted together in a single assessment at such a spatial administrative unit along a coastal area. Consequently, the CVI results obtained in this study are much more robust than any previous CVI studies.

**Figure 8:**
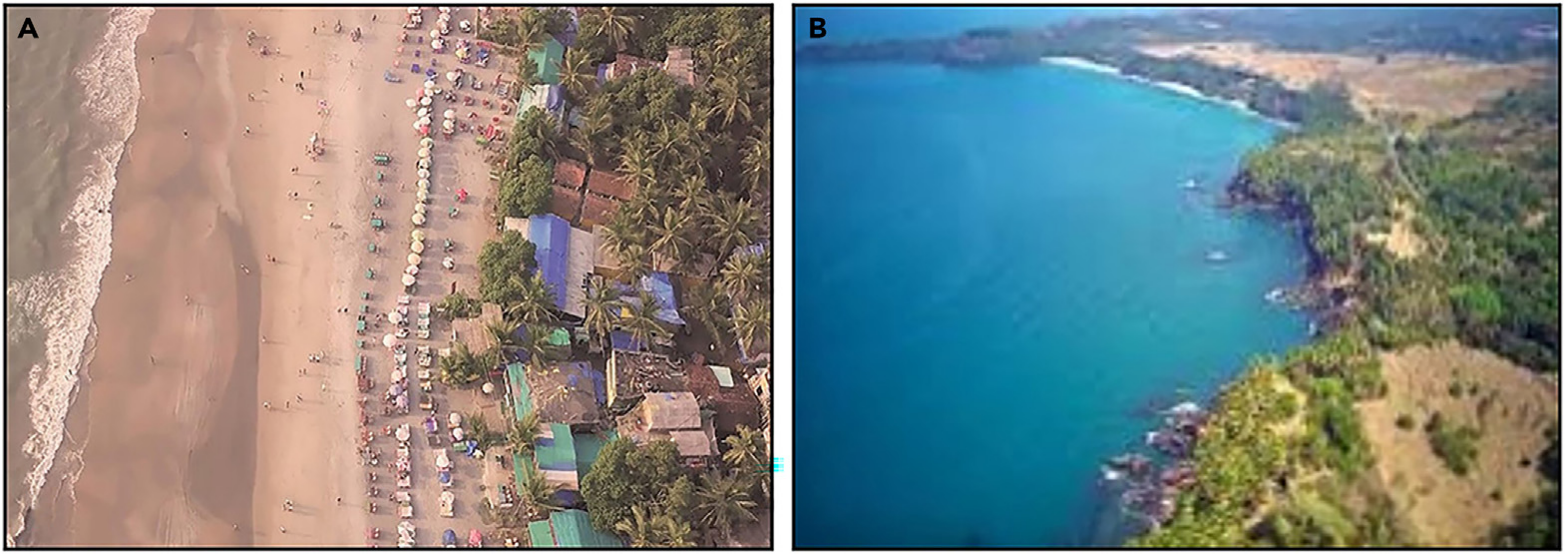
Drone images of coastal stretches of the study area (A) Sandy coast and (B) cliffed coast.

**Figure 9:**
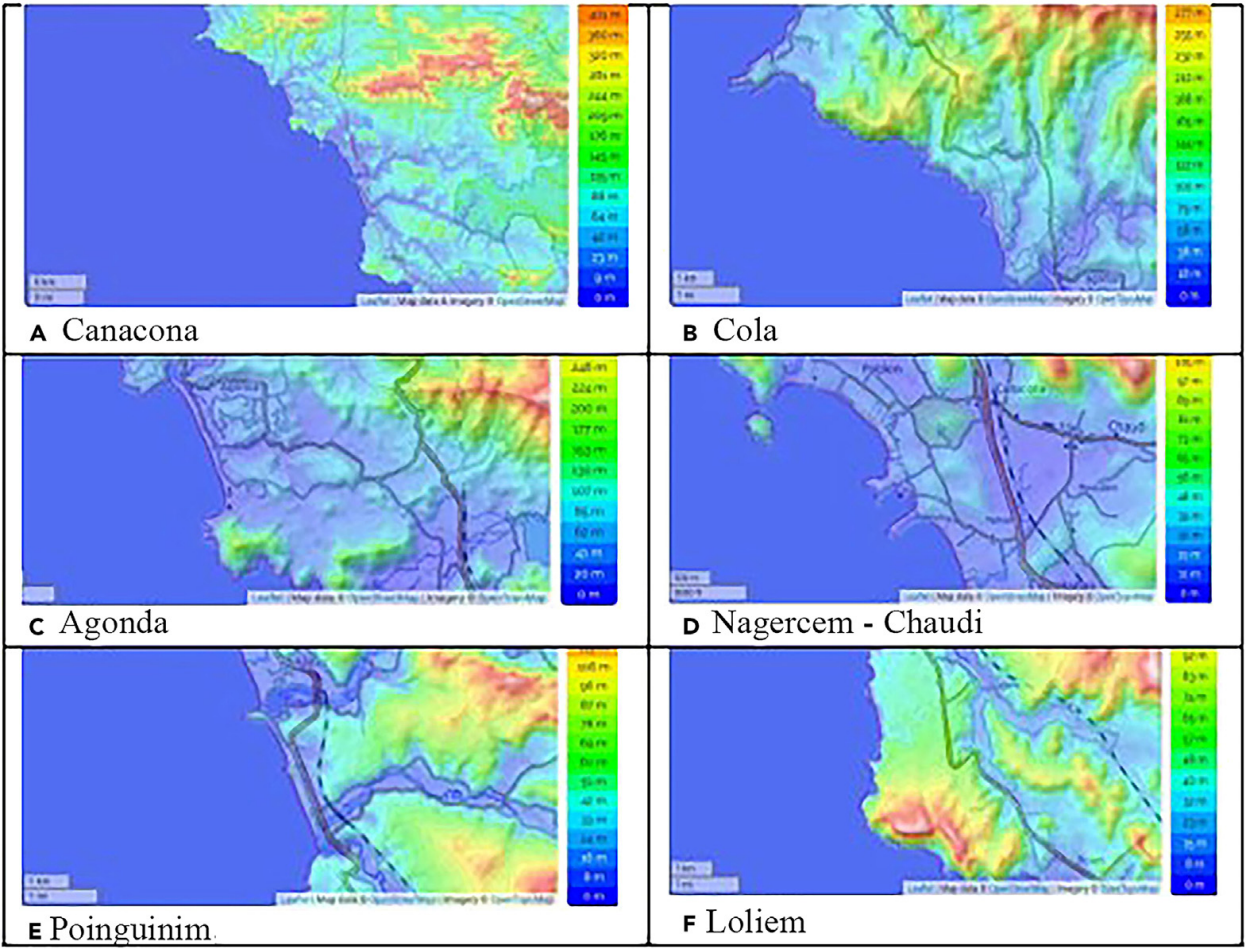
Topographic maps showing differences in elevation along the coastline (A) Canacona (Entire Study Area), (B) Cola, (C)Agonda, (D) Nagercem-chaudi, (E) Poinguinim, and Loliem (F).

Kunte et al.^7^ had ranked the taluka of Quepem and Canacona as the least vulnerable to erosion due to the presence of rocky cliffs, exposed rocks, and mesas. They also have low population density and do not attract many tourists.^7^ In contrast, the present study revealed that Agonda and Nagercem-Chaudi are the most vulnerable coastal villages in Canacona. They have low-lying topography, such as a high proportion of sandy beach stretch (Figure 8) and low regional elevation (Figure 9). The land use/land cover has drastically changed in the last two decades due to increased tourist activity. Due to the availability of low-cost accommodation in comparison to North Goa beaches, tourism has thrived here. To cater to this need, haphazard development with congested roads along the estuarine plain has resulted in high social vulnerability scores (Figure 7). Loliem, for its part, is entirely on the opposite side of the vulnerability scale due to its high regional elevation and rocky coast (Figure 8). Socio-economically, it has low population density, open roads, less traffic congestion, less population to serve, etc.

Therefore, local-level variables like type of coastline, nearshore bathymetry (Figure 3), regional elevation, high population and tourist density, and settlement pattern are crucial in defining coastal vulnerability at the most localized level. In addition, it has come to light that the population centers located along the coast that have special administrative and commercial infrastructure generally serve a larger population from the nearby centers, which subsequently leads to the development of other public amenities such as; high-density commercial areas, transportation facilities, residential projects, etc. As a result, they attract more people who prefer to live there due to all these amenities, and such migration puts more pressure on the land resources. Thus, when a population center discussed here (Nagercem-Chaudi) is located in geological settings (low elevation, gentle slope, sandy coast, sparse vegetation along the coastline etc.) then the vulnerability would increase manifolds compared to a town with no administrative liabilities (Cola), and which does not attract population due to this specific reason.

These localized CVI variables can differentiate one village from another and make a strong case for conducting village-level coastal vulnerability studies in the future.

### Limitations of the study

While this study provides valuable insights into coastal vulnerability at the village level in Canacona, Goa, it is important to acknowledge certain limitations. First, the assessment primarily relies on physical, geological, and socio-economic variables. While these variables are important factors in assessing vulnerability, cultural and historical aspects also play a significant role. Neglecting these dimensions can lead to an incomplete understanding of the complex factors contributing to vulnerability, as cultural and historical aspects can influence how communities perceive and respond to risks. The CVI developed in this study may not capture all nuanced factors contributing to vulnerability.

Using ordinal scales in vulnerability assessments, as described in the chosen methodology based on USGS guidelines, introduces a level of subjectivity into the process. Ordinal scales involving ranking variables can be useful, but they allow for some degree of interpretation and subjectivity, particularly when assigning weights to different factors. This subjectivity could influence the final CVI values, impacting the accuracy of the vulnerability assessment's outcomes.

Furthermore, the study emphasizes vulnerability’s physical and socio-economic dimensions, neglecting the temporal aspect. Coastal vulnerability is dynamic due to evolving climatic conditions, human interventions, and other changes in management resulting from policy changes.

Lastly, while incorporating a hydrological modeling component, the validation approach has its assumptions. While valuable, the “bathtub” model simplifies complex coastal processes, and the eight-sided HC rule might oversimplify the actual hydrological dynamics.

Despite these limitations, this study serves as a pioneering effort in assessing coastal vulnerability at the local level. Future research could benefit from a more comprehensive inclusion of cultural and historical factors, a standardized ranking system, and a longitudinal analysis to enhance the precision and applicability of vulnerability assessments in coastal regions.

### Conclusion

Concerns about climate change have led to a growing body of research on coastal vulnerability to SLR.^10^ Few studies have included socioeconomic indicators in their coastal vulnerability assessment.^18^ The work presented here goes beyond what has been achieved in India but remains only a first step toward a comprehensive vulnerability assessment. The present study uses 21 variables to compute the CVI, based on 10 physical and geological variables comprising the PVI and 11 socio-economic variables comprising the SoVI. Even though one of the first CVI studies conducted by Gornitz et al. (1991) suggested that population density should be considered as a vital risk variable, few studies have included this parameter or went beyond this parameter to conduct an assessment of social vulnerability. Social vulnerability variables play a significant role in defining a region's vulnerability. The results have shown high spatial variability in the vulnerability of the Canacona coastline due to its varied topography and differences in social patterns across the villages, along the coast of the taluka, which can significantly influence coastal vulnerability. In Goa, hazard-related information is collected at various levels, i.e., the district, taluka, and village. The various development and environmental decisions taken by the Chief Minister and his committee and District magistrates are based on this information. The decisions are implemented by the village panchayat (which has a stronghold at the taluka level administration). The current village-based vulnerability index study and the resulting map provide valuable data to decision-makers by depicting areas most vulnerable coastal hazards.

## STAR★Methods

### Resource availability

#### Lead contact

Further information and requests for resources should be directed to and will be fulfilled by the lead contact, Dr. Bruno Damásio (bdamasio@novaims.unl.pt).

#### Materials availability

The data related to Physical variables incorporated in the current study are open access which have been taken from various scientific institutions working under Government of India.

The data related to Socio-economic variables incorporated in the current study are open access which have been taken from the Census of India, 2011.

The current study did generate geospatial and field data for the physical variables (Vegetation behind the beach, regional elevation, percentage of sandy coast and sand dune density).

#### Data and code availability

The current study has adopted an index based approach in which the already available open sources datasets related to coastal vulnerability assessment have been taken primarily from the government and scientific institution working under the Government of India. The data are publicly available as of the date of publication. Complete sources and references are listed in the the main reference list respectively. There is no code associated with this article.

Any additional information required to reanalyze the data reported in this paper is available from the lead contact upon request.

### Method details

This study uses a modified version of the CVI methodology first published by the United States Geological Survey (USGS),^11^^,^^12^^,^^13^ which assesses coastal vulnerability on the basis of six physical and geological variables. In the current study, a Physical Vulnerability Idnex (PVI) is computed using 10 variables, including the six variables from the original USGS index, i.e.,: rate of relative sea-level change, rate of shoreline change (erosion or accretion), mean tidal range, significant wave height, coastal slope and geomorphology, plus coastal regional elevation and other variables that have not yet been incorporated in CVI along the coast of India, that is percentage of sandy coast, sand dune density and vegetation behind the beach, or their use has yet been limited: plausible storm surge height. These latter thee variables were added because local morphological factors and vegetation can influence the degree of vulnerability. The SoVI, for its part, comprises 11 social variables; Village population density, Road density, Total settlement area under village (%), Water supply through pipeline (%), Literacy rate (%), Dependent population (%), Female population (%), Polulation between (0–6 years) %, Tourist density, Settlement area under 500m HTL and Settlement area under 200m HTL.

Different methods are used to determine the values of the variables to comprising an index. The USGS methodology^11^^,^^12^^,^^13^ consists of ranking each variable on an ordinal scale from one to five. Most studies, worldwide, have followed this ranking procedure but without necessarily using the same range for the ranking of the risk variables as no standards exist to determine what values should be considered as low, medium, or high when a three-category scale is used, for instance, nor is it the case when five ranking categories are used. Boruff et al.,^10^ following the USGS methodology, ranked the variables as low, moderate, and high, while Kumar and Kunte (2012) classified the coastline of Chennai into three vulnerability categories. Likewise, this study has followed a linear scale from 1 to 3 to increase vulnerability from low to moderate to high (Table 2). With the help of expert knowledge, all the 21 variables were weighted accordingly (Table 3). Weightage score of 3 physical variables (rate of relative SLR, mean tidal range, and significant wave) is taken from Kunte et al.^7^ as changes in those variables along 42 km study area of Goa coastline are marginal. Sand-dune density and Vegetation behind the beach along the Canacona coastline were ranked qualitatively based on the field observations taken by experts.

Later, to validate the results of the Coastal Vulnerability Index, a hydrological modeling approach was implemented to assess the coastal vulnerability associated with potential coastal hazards. The study utilized a modified "bathtub" model in conjunction with local Digital Elevation Models (DEMs) to outline the areas susceptible to future sea-level rise (SLR) inundation. In contrast to the conventional "bathtub" model, which assumes that land elevation below projected sea levels will be submerged, the modified model incorporates HC. This feature enables the model to exclude areas from inundation that are disconnected from open water.

Three plausible compound coastal flooding inundation scenarios consisting of sea level rise (SLR), higher high water at spring (HHWS) and storm surge (SS) have been used to determine the extent of inundation along the coast which is directly proportional to the coastal vulnerability in the respective area. The 30-m freely available SRTM DEM was retrieved from the NASA earth explorer for the study area (See Table 8).

### Quantification and statistical analysis

The weightage of each one of the 21 variables have been taken for calculation of indices as per the procedure described below.
